# Release kinetics and mitogenic capacity of collagen barrier membranes supplemented with secretome of activated platelets - the in vitro response of fibroblasts of the periodontal ligament and the gingiva

**DOI:** 10.1186/s12903-017-0357-6

**Published:** 2017-03-21

**Authors:** Eva-Maria Mozgan, Michael Edelmayer, Klara Janjić, Manuela Pensch, Michael B. Fischer, Andreas Moritz, Hermann Agis

**Affiliations:** 1 Department of Oral Surgery, School of Dentistry, Medical University of Vienna, Sensengasse 2a, 1090 Vienna, Austria; 2 Department of Conservative Dentistry and Periodontology, School of Dentistry, Medical University of Vienna, Sensengasse 2a, 1090 Vienna, Austria; 3 Department of Blood Group Serology and Transfusion Medicine, Medical University of Vienna, Sensengasse 2a, 1090 Vienna, Austria; 4 Center for Biomedical Technology, Danube University Krems, Dr.-Karl-Dorrek-Straße 30, Krems, 3500 Austria; 5 Austrian Cluster for Tissue Regeneration, Donaueschingenstr. 13, 1200 Vienna, Austria

**Keywords:** Platelets, Collagen barrier membranes, Tissue engineering, Guided bone regeneration, Thrombocytes

## Abstract

**Background:**

Platelet preparations can stimulate the healing process and have mitogenic properties. We hypothesized that collagen barrier membranes (CBM), clinically used in guided bone regeneration and guided tissue regeneration, can serve as carriers for platelet secretome.

**Methods:**

Secretome was generated from washed platelets and unwashed platelets (washed/unwashed PSEC) and lyophilized onto CBM. Overall appearance of CBM was evaluated by scanning electron microscopy. The impact of PSEC on cell attachment was measured based on fluorescence microscopy with DiI-labeled cells. To assess the release kinetics, supernatants of CBM were collected and medium was replaced at hour 1–48. The mitogenic effect was evaluated with periodontal fibroblasts. Furthermore, the release of total protein, platelet-derived growth factor (PDGF)-BB, and transforming growth factor (TGF) β1 was measured.

**Results:**

CBM overall appearance and cell attachment was not modulated by PSEC. Supernatants taken after one hour induced a mitogenic response in fibroblasts and showed the highest levels of total protein, TGFβ1 and PDGF-BB. These effects decreased rapidly in subsequent supernatants. While supernatants of CBM loaded with unwashed PSEC induced a stronger mitogenic response than supernatants of CBM loaded with washed PSEC this difference between the PSEC preparations was not observed when cells were seeded on 48–hours-washed CBM.

**Conclusions:**

CBM release platelet-derived factors in continuously declining release kinetics.

## Background

In guided tissue regeneration collagen barrier membranes (CBM) are used as scaffolds to provide structural support for repair cells and prevent the ingrowth of soft tissue into periodontal and bone defects [1–3]. The success of this approach depends on the regenerative capacity of the respective tissue. When healing is compromised, therapeutic approaches based on biologicals can support regeneration. CBM represent a promising carrier for bioactive molecules that stimulate the healing capacity as they are placed in the interface between soft and hard tissue defects where they can release factors and pharmaceuticals to target cells of both tissues [4, 5]. Platelets have been investigated for their capacity to stimulate oral tissue regeneration based on their key role in the wound healing cascade and their capacity to modulate inflammation [6–9].

Wound healing and bone regeneration are initiated by the formation of a blood clot. In these blood clots, activated platelets release a myriad of growth factors that stimulate migration and proliferation of repair cells [10–12]. Among these factors are platelet-derived growth factor (PDGF) isoforms and transforming growth factors- β1 (TGFβ1). PDGF-BB activates all three PDGF receptor isoforms: PDGFRαα, PDGFRαβ, and PDGFRββ. Based on its potent activity, PDGF-BB was approved for the clinical application in periodontal tissue regeneration [13, 14]. TGF-β1 induces matrix production and cell proliferation and can bind to collagen [15–21]. The finding of mitogenic effects of activated platelets induced the development of regenerative strategies based on platelet concentrates [6–9, 22].

Platelet-rich plasma and platelet-rich fibrin can stimulate bone and periodontal ligament regeneration based on the high growth factor content of platelets [23]. Although platelet preparations, such as platelet-rich plasma and platelet-rich fibrin have been intensively studied, their effect on oral tissue regeneration and the underlying cellular activities is not entirely clear. Recently, cell-free approaches based on cell secretome have been proposed for regenerative purposes [24]. In vitro studies have shown that the secretome of activated platelets is mitogenic and that the preparation protocol modulates their effect on cell function including differentiation of osteoblasts and osteoclasts [25, 26].

Collagen can bind growth factors and therefore clinically applied CBM represent a promising carrier for the secretome of activated platelets [15, 16, 19–21]. In vitro studies have shown that the preparation protocol of the secretome can modulate the effect of bone cells. In particular the presence of serum components in the platelet preparation can have a high impact as the secretome of washed platelets and unwashed platelets has different effects. How the presence of serum components affect the release kinetics from CBM is unknown. Here we aimed to reveal the mitogenic capacity of CBM loaded with two different platelet preparations, generated by an established protocol [25, 26]: Secretome of washed platelets (washed PSEC) and secretome of unwashed platelet preparations (unwashed PSEC) Both preperations can stimulate proliferation [25, 26]. We evaluated if loading with washed and unwashed PSEC changes the morphology and cell attachment on the membrane. An established bioassay was applied to assess the mitogenic activity released from the CBM over time [27]. In addition, we assessed the release kinetics directly based on total protein, PDGF-BB, and TGFβ1. Finally, we evaluated if the bound PSEC activity differs between CBM loaded with washed and unwashed PSEC.

## Methods

### Preparation of washed PSEC and unwashed PSEC

**Fig. 1:**
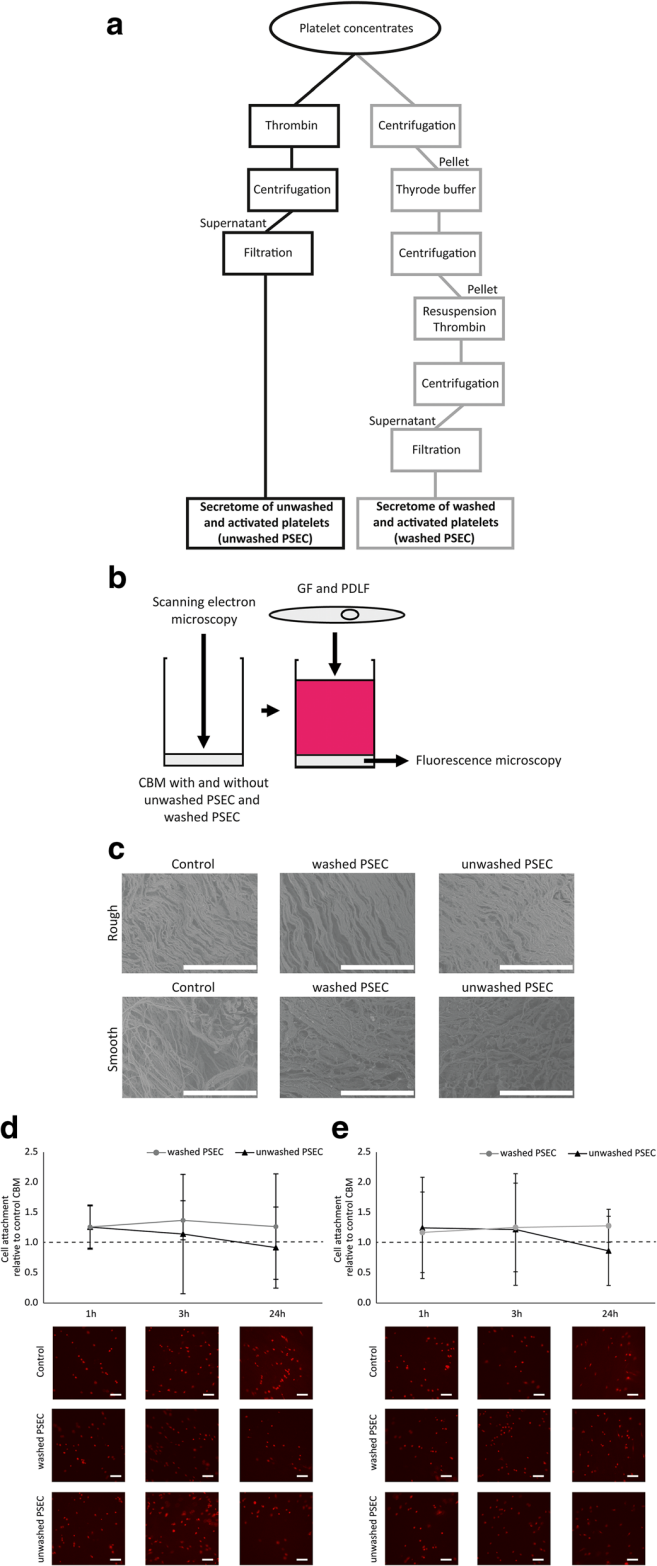
Unwashed PSEC or washed PSEC was loaded onto CBM and membrane morphology and cell attachment was evaluated. Platelet concentrations were divided into two equivalent aliquots and processed as described to generate unwashed PSEC and washed PSEC (**a**). CBM were soaked with unwashed PSEC or washed PSEC, immediately frozen and lyophilized. Morphology of CBM loaded with unwashed PSEC or washed PSEC was evaluated with scanning electron microscopy and cell attachment was assessed with fluorescence microscopy (**b**). For scanning electron microscopy images were taken at a 500-fold magnification (The white bar represents 200 μm) (**c**). An in vitro bioassay was used to reveal the attachment kinetics of GF and PDLF to CBM: PDLF (**d**) and GF (**e**) were pre-labeled with DiI and seeded on CBM loaded with unwashed PSEC or washed PSEC. The membranes were washed and images were taken 1, 3, and 24 h and the attached cells were counted. The *data points* show the mean ± standard deviation relative to the untreated control (*n* = 3). The *dashed line* represents the levels of the untreated control

Platelet concentrates were generated at the Clinic for Blood Group Serology and Transfusion Medicine, Medical University of Vienna (The protocol was approved by the Ethics Committee of the Medical University Vienna) by single donor platelet apheresis using the Trima Accel® Automated Blood Collection System (TerumoBCT, Lakewood, CO). Platelets were stored under constant agitation in platelet additive solution SSP+ (Macropharma, Tourcoing, France) containing 20–30% plasma and forwarded for scientific use after a period of up to 6 h post-production. To generate washed and unwashed PSEC, concentrates were divided into 2 units (Fig. 1a): For unwashed PSEC, platelet concentrates were activated with 2 U human thrombin/mL. After coagulation the preparations were centrifuged. For washed PSEC the platelet concentrates were centrifuged, resulting in a platelet pellet. The platelet pellet was washed with Tyrode buffer, resuspended in an equivalent volume of serum-free medium and activated with 2 U human thrombin/mL as described above. The supernatant was obtained after centrifugation. All supernatants were sterile-filtered and stored in aliquots at -80 °C. The stock solutions represent platelet concentrations of approximately 1 x 10^9^ platelets/mL.

### Loading of CBM with washed PSEC and unwashed PSEC

CBM (BioGide®) were provided by Geistlich Pharma AG (Wolhusen, Switzerland). In brief, CBM were cut into specimens of 5 mm in diameter. These specimens were soaked with washed and unwashed PSEC and frozen at -80 °C. Lyophilization was performed using a Freeze dryer ALPHA 1–2 LDplus (Martin Christ, Osterode am Harz, Germany) at the Center for Physiology and Pharmacology, Medical University of Vienna.

### Scanning electron microscopy evaluation of CBM

The overall appearance of the rough and smooth side of the CBM was evaluated by scanning electron microscopy (SEM). Therefore, the lyophilized CBM were observed using the TM-1000 table top system (Hitachi, Tokyo, Japan). The specimens were mounted on an aluminium stub and images were captured from both sides of the membranes (rough and smooth) at 15,000 V accelerating voltage at a 500-fold magnification.

### Cell culture

After informed consent, fibroblasts were prepared from extracted third molars (The protocol was approved by the Ethics Committee of the Medical University Vienna). The donors had no previous history of dental inflammation. Fibroblasts were isolated as described previously [28]. In brief: Gingival fibroblasts (GF) were prepared from the soft tissue of the gingiva attached to the tooth neck. Periodontal ligament fibroblasts (PDLF) were prepared from the soft tissue attached the tooth root. GF and PDLF were expanded and cultivated in α-minimal essential medium (Invitrogen Corporation, Carlsbad, CA), supplemented with 10% fetal calf serum (FCS, PAA Laboratories, Linz, Austria) and antibiotics (Invitrogen Corporation) at 37 °C, 5% CO_2_ and 95% humidity.

### Cell attachment assay

Cells were labeled with DiIC12(3) (BD Biosciences, Bedford, MA, USA) according to manufacturer’s instructions. 30,000 cells/cm^2^ were seeded onto the smooth side of the CBM. After 1, 3, and 24 h the membranes were harvested and unattached cells were washed off the membranes with phosphate buffered saline. Membranes were immediately evaluated in an upright fluorescence microscope (Nikon Eclipse E 800 M microscope; Nikon Instruments Europe B.V., Badhoevedorp, Netherlands). Images were taken, blinded and cells were counted by an independent investigator using ImageJ (NIH, USA).

### Release of washed and unwashed PSEC from CBM

Supernatants from CBM were generated by adding 150 μL of serum - free α-minimal essential medium (Invitrogen Corporation) supplemented with antibiotics to one CBM specimen. Supernatants were collected and replaced with fresh medium at hour 1, 3, 6, 24, and 48. These supernatants were subjected to bioassays with GF and PDLF and bicinchoninic acid (BCA) assays, and immunoassays for PDGF-BB and TGFβ1.

### ^3^[H]thymidine incorporation assay

Cells were plated at 50,000 cells/cm^2^ in 96 well plates. The following day medium was changed to serum - free α-minimal essential medium supplemented with antibiotics. Cells were incubated for 24 h with supernatants of CBM. To assess the effect on proliferation, cells were pulse-labeled with ^3^[H]thymidine (0.5 μCi/well, Hartmann Analytic, Braunschweig, Germany) for the last 6 h of exposure. The plates were assessed by liquid scintillation counting (Packard, Meriden, CT) and data was normalized to cells that were exposed to supernatants of unloaded CBM.

### BrdU assays

Cells in serum free α-minimal essential medium supplemented with antibiotics were plated at 30,000 cells/cm^2^ onto the CBM loaded with washed and unwashed PSEC. Cells were cultured for 24 h on the CBM and pulse-labeled with BrdU for the last 6 h of exposure. The assay was performed according to the protocol of the manufacturer and data was normalized to cells that were exposed to unloaded CBM.

### BCA assay

Supernatants of the CBM were evaluated using the BCA assay (Thermo Scientific, Rockford, IL, USA) according to the instructions of the manufacturer. In brief: BCA A and B were mixed 1:50 and 100 μL were added per 10 μL of the 1:10 diluted sample. After 30 min at 37 °C absorption was assessed in a photometer at 550 nm.

### Immunoassays for PDGF-BB

To evaluate the release of platelet-derived factors from the CBM PDGF-BB was assessed in the supernatants by immunoassays. Supernatants from CBM were harvested and subjected to Enzyme-linked immunosorbent assay (ELISA) for PDGF-BB. The measurements were performed using the PDGF-BB ELISA kit (Peprotech, Rocky Hill, NJ, USA) according to the description of the manufacturer.

### Immunoassays for TGFβ1

To assess the release of TGFβ1 from the CBM the supernatants were assessed using the respective immunoassay. Supernatants of CBM were harvested and subjected to ELISA for TGFβ1. The measurements were performed using the Human TGF beta 1 ELISA kit (Cohesion Biosciences, London, UK) according to the description of the manufacturer.

### Statistical analysis

Data are given as mean ± standard deviation and area under the curve (AUC) calculations were performed in indicated experiments. Data were compared by ANOVA and paired t-test. Significance was assigned at *p* < 0.05 level.

## Results

### Loading CBM with washed and unwashed PSEC neither modulates morphology nor suppresses cell attachment

CBM were loaded with washed and unwashed PSEC (Fig. 1a, b). The here applied CBM has a smooth and a rough side. While the smooth side is the side facing the soft tissue, the rough side is considered to support bone formation. To reveal if loading with washed and unwashed PSEC changes membrane overall appearance of the rough and smooth side, the membranes were evaluated using SEM. No difference in morphology was observed between the CBM loaded with washed and unwashed PSEC (Fig. 1c). The smooth side shows an even surface structure after lyophilization, whereas the rough side shows an irregular structure. To reveal if supplementation of CBM with unwashed and washed PSEC modulates cellular attachment kinetics to the membranes, DiI-labeled GF and PDLF were seeded onto the smooth side of the CBM which is the side facing the soft tissue when applied clinically (Fig. 1d, e). Cells appeared round on the CBM after 1 h. Over time cells became more flat as suggested by a fibroblast-like morphology. However, there was no significant difference between the numbers of fibroblasts attached to unwashed PSEC-loaded, washed PSEC-loaded, and the unloaded CBM after 1 h, 3 h, and 24 h of incubation. Overall, GF and PDLF behaved similar. These data suggest that loading CBM with unwashed and washed PSEC does not hinder cell attachment to the membranes.

### Supernatants of unwashed PSEC-loaded CBM show a stronger mitogenic effect than washed PSEC-loaded CBM

**Fig. 2:**
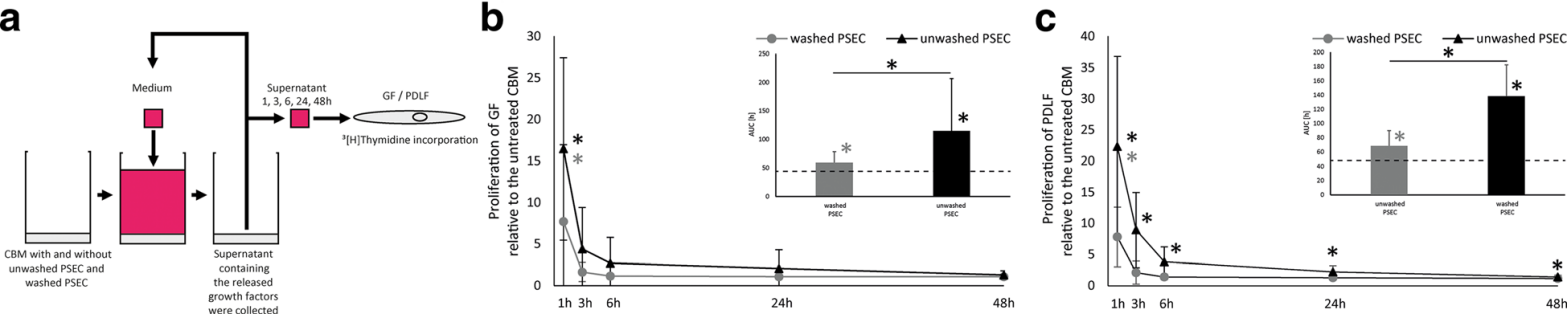
Mitogenic capacity of supernatants from CBM loaded with unwashed PSEC and washed PSEC. An in vitro bioassay was used to reveal the release kinetics of platelet-released supernatants of CBM: Supernatants of CBM loaded with unwashed PSEC and washed PSEC were collected at hour 1, 3, 6, 24, and 48 (**a**). The supernatants were subjected to cell cultures where their capacity to stimulate proliferation was assessed by ^3^[H]thymidine incorporation into GF (**b**) and PDLF (**c**). The *data points* show the mean ± standard deviation relative to the control CBM. For proliferation of GF *n* = 10 and for proliferation of PDLF *n* = 12. The *dashed line* represents the levels of the control CBM. The bar diagrams represent the AUC. * *p* < 0.05

To assess the released mitogenic activity of washed PSEC and unwashed PSEC-loaded CBM, GF and PDLF were incubated with supernatants of the membranes taken at hour 1, 3, 6, 24, and 48 (Fig. 2a). Our data show that supernatants of washed PSEC-loaded and unwashed PSEC-loaded membranes stimulate proliferation of GF and PDLF (Fig. 2b, c). Supernatants generated from washed PSEC and unwashed PSEC-loaded CBM induced an increase in the first hour in proliferation in GF and PDLF. The capacity to enhance proliferation rapidly decreases in the subsequent supernatants of both membrane preparations. Supernatants generated at hour 6 from CBM loaded with washed and unwashed PSEC induced a 1.15-fold and 2.70-fold increase in proliferation of gingival fibroblasts, respectively. Supernatants generated from washed and unwashed PSEC-loaded CBM at hour 6 induced a 1.42-fold and 3.84-fold increase in proliferation of periodontal ligament fibroblasts, respectively. These data suggest that CBM loaded with washed and unwashed PSEC rapidly release platelet-derived factors. Calculation of the AUC revealed that supernatants from CBM loaded with unwashed PSEC have a higher mitogenic capacity than supernatants of CBM loaded with washed PSEC. Taken together unwashed PSEC-loaded CBM show a stronger mitogenic effect than washed PSEC-loaded CBM.

### Unwashed PSEC-loaded CBM release more protein, PDGF-BB, and TGFβ1 than washed PSEC-loaded CBM

**Fig. 3:**
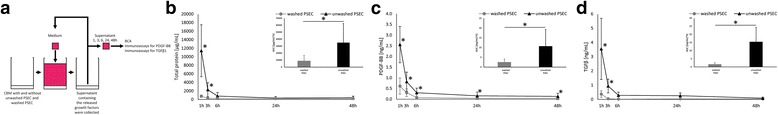
Release kinetics of total protein, PDGF-BB, and TGFβ1 form CBM loaded with unwashed PSEC and washed PSEC. To reveal the release kinetics of platelet released supernatants of CBM we measured the total protein levels, PDGF-BB, and TGFβ1: Supernatants of CBM loaded with unwashed PSEC and washed PSEC were collected at hour 1, 3, 6, 24, and 48 (**a**). The supernatants were assessed with immunoassays for total protein concentration (**b**), PDGF-BB (**c**), TGFβ1 (**d**). The *data points* show the mean ± standard deviation relative to the untreated control. For total protein *n* = 8, for PDGF-BB *n* = 14, for TGFβ1 *n* = 6. The bar diagrams represent the AUC * *p* < 0.05

Next we assessed the release of platelet-derived factors by measuring total protein, PGDF-BB, and TGFβ1 in the supernatant of washed and unwashed PSEC-loaded CBM, respectively (Fig. 3a). Measurement of total protein release revealed that the protein concentration in supernatants of CBM loaded with washed and unwashed PSEC in the first hour were 768 μg/mL and 11423 μg/mL, respectively (Fig. 3b). The protein content declined in the subsequent supernatants taken at hour 3, 6, 24, and 48. Evaluation of growth factor release based on PDGF-BB and TGFβ1 showed similar kinetics (Fig. 3c, d). The highest PDGF-BB concentration was observed in supernatants taken at hour 1. 0.61 ng/mL and 2.56 ng/mL PDGF-BB were observed in the supernatants of washed and unwashed PSEC-loaded CBM, respectively. Also the highest TGFβ1 levels were found in the supernatants taken at hour 1. 0.37 ng/mL and 3.54 ng/mL TGFβ1 were observed in the supernatants of washed and unwashed PSEC-loaded CBM, respectively. We found that the majority of growth factors was released within the first 6 h. Unwashed PSEC-loaded CBM release more protein, PDGF-BB, and TGFβ1 than washed PSEC-loaded CBM. This was further supported by calculations of the AUC.

### CBM can maintain a mitogenic activity after 48 h washing

**Fig. 4:**
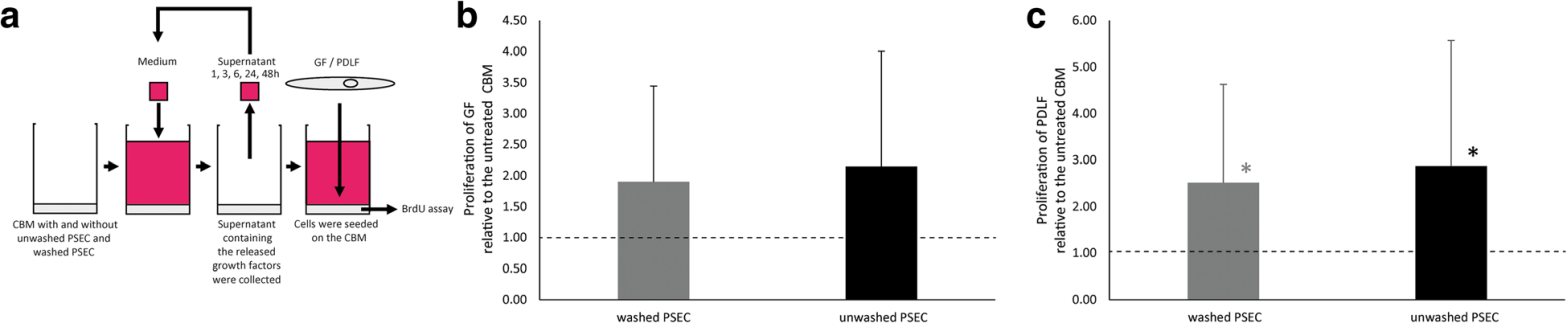
Mitogenic capacity of CBM loaded with unwashed PSEC and washed PSEC and after the 48 h washing procedure. An in vitro bioassay was used to assess if any mitogenic capacity is retained by the membrane after the 48 h of washing: CBM loaded with unwashed PSEC and washed PSEC were repeatedly washed following the same protocol as for the release kinetics studies at hour 1, 3, 6, 24, and 48 to ensure that the membranes have released the growth factors (**a**). GF (**b**) and PDLF (**c**) were seeded onto these membranes. Proliferation was quantified by BrdU assays. The *bars* represent the mean ± standard deviation relative to the control CBM (*n* = 18). The *dashed line* represents the levels of the control CBM. * *p* < 0.05

To assess if the PSEC-loaded CBM retain their mitogenic activity absorbed to the CBM, fibroblasts were seeded onto the loaded CBM that were washed for 48 h as described above (Fig. 4a). Proliferation of GF seeded onto the CBM loaded with washed PSEC and unwashed PSEC were 1.90-fold and 2.14-fold of the unloaded CBM, respectively (Fig. 4b). Proliferation of PDLF seeded onto washed and unwashed PSEC-loaded CBM was 2.52-fold and 2.87-fold of the unloaded CBM, respectively (Fig. 4c). While this increase reached the level of significance in PDLF it was not significant in GF. Our data suggest that CBM loaded with washed and unwashed PSEC can retain part of their mitogenic capacity also after 48 h washing procedure absorbed on the CBM surface.

## Discussion

In guided bone regeneration and guided tissue regeneration CBM are used as scaffolds and barriers to support the healing process [1–3]. Research has focused on optimizing the material and structural properties. Recently, strategies that use these membranes as carriers for bioactive molecules have been proposed [5, 16, 20]. Here, we follow an approach where CBM are used as carrier for platelet-derived factors. This approach is based on the concept that CBM can bind growth factors [16, 20]. We found that CBM loaded with washed and unwashed PSEC induce a mitogenic response indicating a continuous declining release of PSEC from the CBM. However, they also retain mitogenic capacity adsorbed to the surface of the CBM.

Cell attachment to the surface of the CBM is an integral step in the early phase of the healing process. Therefore, it is crucial to understand how processing affects the morphology of the membrane and cell attachment. We observed attachment at hour 1, hour 3, and hour 24 to assess in particular early attachment, based on previous studies of cell attachment on titanium surfaces [29]. In our setting, where we lyophilized the CBM with washed and unwashed PSEC, neither appearance of the membrane nor the numbers of cells attached to the membranes were compromised.

Supernatants of CBM loaded with washed and unwashed PSEC taken after one hour increased ^3^[H]thymidine incorporation an indicating proliferation. The effect on cell proliferation decreased rapidly in subsequent supernatants taken after 3, 6, 24, and 48 h. These results are in line with the release kinetics of total protein, PDGF-BB, and TGFβ1, and our previous studies on the release of prolyl hydroxylase inhibitors from CBM [5]. Protein levels, PDGF-BB levels, and TGFβ1 levels were high in the first hour and decreased in supernatants which were taken later suggesting that the majority of growth factors was washed out in the first hours of incubation. Overall, supernatants of unwashed PSEC-loaded CBM showed a stronger stimulation of cell proliferation than washed PSEC-loaded membranes and the growth factor concentration in the supernatants of unwashed PSEC-loaded CBM was higher than in supernatants of CBM loaded with washed PSEC, suggesting that loading the membranes with unwashed PSEC might be more effective than with washed PSEC. These data are in line with direct measurements of the growth factor release.

In this study we used washed PSEC and unwashed PSEC which contains PDGF-BB and TGFβ1. Both growth factors show similar release kinetics in this model. Recombinant human PDGF-BB with beta-tri calcium phosphate is applied in clinic to stimulate periodontal regeneration by enhancing cell proliferation and cell attachment via activation of all isoforms of the cellular PDGF receptor, namely PDGFRαα, PDGFR-αβ, and PDGFR-ββ [30–32]. However, recombinant human PDGF-BB is rapidly released from the beta-tri calcium phosphate within 90 min [33]. Noncross-linked bovine type I collagen loaded with PDGF-BB or GDF-5 improved bone healing and release of recombinant human PDGF-BB over several days [34, 35]. The membrane was loaded by soaking for 1 h in the growth factor solution. The CBM used in our study is a noncross-linked collagen type I/III which was loaded with washed and unwashed PSEC by lyophilization. The differences in the release profiles might be due to the different preparation and loading technique as well as the different types of membranes used. Our results from the washed PSEC and unwashed PSEC loaded CBM show a burst-like release of proteins, PDGF-BB and TGFβ1 within the first 6 h which is in line with the mitogenic effects of the supernatants in the bioassays.

Based on these findings one might suspect that the PSEC-loaded CBM release their entire mitogenic capacity within the 48 h observation period. However, our results show increased proliferation of PDLF that have been seeded onto the PSEC-loaded CBM after the 48 h period. The elevated proliferation levels of GF did not reach the level of significance. Taken together these results suggest that CBM can retain part of their mitogenic capacity. This finding is not surprising as CBM can bind TGFβ activity [15, 16, 19–21]. Thus, it might be possible that growth factors of PSEC remain bound to the collagen membrane where they can stimulate cell proliferation. The two platelet preparations, washed and unwashed PSEC, did not show any difference in regard to the proliferation of cells seeded onto the CBM. Our findings suggest that while there is a continuous declining release CBM can adsorb PSEC activity.

It is unclear if this feature is a specific capacity of the CBM used in this study. Here we used a commercially available CBM (BioGide®) as carrier for the washed and unwashed PSEC. This CBM is composed of type I and type III collagen of porcine origin and shows a bi-layered structure [3, 36]. CBM with a different structure might show different release kinetics. Comparison of BioGide® with the single-layered CBM BME-10X composed of bovine collagen I revealed different release kinetics of bisphosphonates from these membranes [4]. Clearly, platelets release other factors besides PDGF-BB and TGFβ1 that induce mitogenic effects, including other PDGF isoforms such as PDGF-AB and other signalling molecules [37]. PDGF-BB is the major PDGF isoform in freeze-dried PRP, therefore we used it as a surrogate marker for the release of platelet-derived signal factors of the CBM in the direct release assays [38]. In the present study, the decrease in PDGF-BB and TGFβ1 release over time was paralleled by a decrease in the overall protein and mitogenic capacity in the supernatants of the PSEC loaded CBM, suggesting that these released growth factors show similar release kinetics.

In our study we chose an in vitro approach to assess the biological activity and release kinetics of supernatants of activated platelets of CBM. The bioassays used to assess the mitogenic capacity of the CBM loaded with washed and unwashed PSEC were based on primary human fibroblasts of the gingiva and the periodontal ligament. One might argue that fibroblasts of the gingiva and the periodontal ligament only partly reflect the in vivo situation, as osteoblasts that are required to form new bone at the defect site were not included in the study. However, results from our previous study show that washed and unwashed PSEC stimulate proliferation of osteoblasts in a similar manner. These data, together with the fact that periodontal fibroblasts and osteoblasts express receptors for PDGF-BB and TGFβ1, indicates that similar effects can be expected on proliferation of osteoblasts and that the applied bioassay based on primary oral fibroblasts represent a viable system to assess the mitogenic capacity of the CBM [39–41]. The present in vitro study is a pre-requisite for further in vivo studies to assess the impact of CBM loaded with supernatants of activated platelets. Freeze-dried PRP-coated collagen scaffolds - while being mitogenic in vitro *-* reduced proliferation in vivo [38]. Horimizu et al. suggested that this is caused by the fibrin gel [38]. In the present study we used supernatants from activated platelets without fibrin. Whether this approach is beneficial compared to PRP requires further preclinical studies. A potential clinical application represents compromised healing. Therefore preclinical studies should be designed to reveal if PSEC loaded CBM can be of benefit in aged individuals or under the impact of diabetes.

## Conclusion

Our results show that CBM can be loaded with washed and unwashed PSEC to induce a mitogenic response. The PSEC-loaded CBM show continuously declining release kinetics of their growth factors and remain a surface-bound source of mitogenic capacity. While the released mitogenic capacity of CBM loaded with unwashed PSEC is stronger than in the CBM loaded with washed PSEC, the membrane associated mitogenic capacity does not differ between the two preparations. Further studies are required to investigate if these in vitro observations translate into enhanced periodontal regeneration in vivo.

## Abbreviations

ANOVA: Analysis of variance
AUC: Area under the curve
BCA: Bicinchoninic acid assay
CBM: Collagen barrier membranes
ELISA: Enzyme-linked immunosorbent assay
GF: Gingival fibroblasts
PDGF: Platelet-derived growth factor
PDLF: Periodontal ligament fibroblasts
PSEC: Platelet secretome
SEM: Scanning electron microscopy
TGFβ1: Transforming growth factor β1
